# Supplementary material for: Upstream open reading frames repress the translation from the iab-8 RNA
Source: PLoS Genet. 2024 Sep 23;20(9):e1011214. doi: 10.1371/journal.pgen.1011214 (PMC11463788; doi:10.1371/journal.pgen.1011214)
Supplement: S1 Text — (DOCX) [file pgen.1011214.s001.docx]

**Supplementary Methods:**

**Transfection protocol:**

S2 cells were transfected using FuGene (Promega, Madison, WI USA) or Effectene (Qiagen, Hilden Germany).

For Fugene: 91uL OptiMEM  was mixed with 9uL plasmids (100ng/uL : 3uL pActGAL4; 3uL pUASmCherry-alphaTubulin; 3uL either pUAS-MSA/Iab8-GFP derivatives).

The mixtures were votexed then 4uL Fugene (without touching borders) were added, mixed gently, incubated 30' at room temperature then added to the cells in a 6-wells plate (10^6 cells in 3mL). After 3 days incubation at 25°C GFP and mCherry was analyzed. For 24 well plate assays, reagents were divided by 3/4 (1uL FuGene and 1uL each plasmid) and around 0.33 X 10^6 cells were placed in 1mL per well.

For Effectene:

Transfections were prepared as follows:

45uL EC buffer, 4uL enhancer,1,5 uL pActGAL4 (100ng/uL),1,5 uL pUASmCherry-alphaTubulin  (100ng/uL) and 1,5 uL pUAS-MSA/Iab8-GFP derivatives (100ng/uL) were vortexed in a tube, then incubated for 5' at RT. After this, 5uL of Effectene was added to the tubes and mixed by gently flicking of the tube. The mixture was left ot rest for 10' at RT, then 56uL of the mix was added dropwise onto 10^6 freshly seeded cells (in 0,5mL)

**Generation of plasmids:**

**pUAS-exon-3-from-msa (referred to as construct exon3)**

-Vector: We digested the pUAST-attB plasmid with the EcoRI RE and purified the

fragment (8.5kb).

-Fragment: We performed a PCR on pUAS-msa-M3-mipep-GFP with the F

UAS_exon3/R UAS-ex8 primers, and we purified the amplified fragment (2,5kb).

Gibson Assembly reaction was performed with the vector and the fragment

followed by a transformation in DH5α thermo-competent cells.

Candidate colonies were screened by digestion using the BamHI RE.

**pUAS-ex1-2-msa (referred to as construct *iab-8*)**

-Vector: We digested the pUAST-attB plasmid with the EcoRI RE and purified the

fragment (8.5kb).

-Fragments:

1-We performed a PCR on pUAS-msa-M3-mipep-GFP with the F EX2-I8_msaex1/

R UAS-ex8 a primers, and we purified the amplified fragment (143bp).

187

2-We performed a PCR on pUAS-iab8-M3-mipep-GFP with the F UAS_ex1-i8 / R

MSAEX1_ex2-i8 primers, and we purified the amplified fragment (1.3kb).

Gibson Assembly reaction was performed with the vector and the fragments

followed by a transformation in DH5α thermo-competent cells.

Candidate colonies were screened by digestion using the BamHI RE.

**pUAS-exon-1 (referred to as construct exon1)**

-Vector: We digested the pUAST-attB plasmid with the EcoRI RE and purified the

fragment (8.5kb).

-Fragments:

1-We performed a PCR on pUAS-iab8-M3-mipep-GFP with the F UAS_ex1-i8/R

EX3_ex1-i8 a primers, and we purified the amplified fragment (785bp).

2-We performed a PCR on pUAS-iab8-M3-mipep-GFP with the F Ex1_ex3/ R UASex8

primers, and we purified the amplified fragment (2.5kb).

Gibson Assembly reaction was performed with the vector and the fragments

followed by a transformation in DH5α thermo-competent cells.

Candidate colonies were screened by digestion using the BamHI RE.

**pUAS-exon-2 (referred to as construct exon2)**

-Vector: We digested the pUAST-attB plasmid with the EcoRI RE and purified the

fragment (8.5kb).

-Fragment: We performed a PCR on pUAS-iab8-M3-mipep-GFP with the F

UAS_ex2/ R UAS-ex8 a primers, and we purified the amplified fragment (3kb).

Gibson Assembly reaction was performed with the vector and the fragments

followed by a transformation in DH5α thermo-competent cells.

Candidate colonies were screened by digestion using the BamHI RE.

**pUAS-30 (referred to as construct 30bp)**

-Vector: We digested the pUAST-attB plasmid with the EcoRI RE and purified the

fragment (8.5kb).

-Fragments:

1-We performed a PCR on pUAS-iab8-M3-mipep-GFP with the F exon8 5’/ R UASex8

a primers, and we purified the amplified fragment (2.3kb).

2-the second fragment was a gBlock fragment mix7 ordered at IDT..

188

Gibson Assembly reaction was performed with the vector and the fragments

followed by a transformation in DH5α thermo-competent cells.

Candidate colonies were screened by digestion using the BamHI RE.

**pUAS-200 (referred to as construct 200bp)**

-Vector: We digested the pUAST-attB plasmid with the EcoRI RE and purified the

fragment (8.5kb).

-Fragment: We performed a PCR on pUAS-iab8-M3-mipep-GFP with the F

UAS_200bp/R UAS-ex8 a primers, and we purified the amplified fragment

(2.7kb).

Gibson Assembly reaction was performed with the vector and the fragments

followed by a transformation in DH5α thermo-competent cells.

Candidate colonies were screened by digestion using the BamHI RE.

**pUAS-262 (referred to as construct 230bp)**

-Vector: We digested the pUAST-attB plasmid with the EcoRI RE and purified the

fragment (8.5kb).

-Fragments:

1-We performed a PCR on pUAS-iab8-M3-mipep-GFP with the F UAS_ex2/R EX3-

delta200 a primers, and we purified the amplified fragment (221bp).

2-We performed a PCR on pUAS-iab8-M3-mipep-GFP with the F DELTA200_ex3/

R UAS-ex8 primers, and we purified the amplified fragment (2.5kb).

Gibson Assembly reaction was performed with the vector and the fragments

followed by a transformation in DH5α thermo-competent cells.

Candidate colonies were screened by digestion using the BamHI RE.

**pUAS-GFP**

-Vector: We digested the pUAST-attB plasmid with the EcoRI RE and purified the

fragment (8.5kb).

-Fragment: We performed a PCR on pUAS-iab8-mipep-GFP with the F UAS_pGFP /

R UAS_gfp a primers, and we purified the amplified fragment (717bp).

Gibson Assembly reaction was performed with the vector and the fragment

followed by a transformation in DH5α thermo-competent cells.

Candidate colonies were screened by digestion using the BamHI RE.

**pUAS-200-GFP/ pUAS-F-GFP**

-Vector: We digested the pGFP plasmid with the EcoRI RE and purified the

fragment (9.2kb).

-Fragment: We performed a PCR on pUAS-iab8-M3-mipep-GFP with the F

UAS_200 / R GFP_200 a primers, and we purified the amplified fragment

(717bp).

Gibson Assembly reaction was performed with the vector and the fragment

followed by a transformation in DH5α thermo-competent cells.

Candidate colonies were screened by digestion using the BamHI RE.

**pUAS-200-ΔATG-GFP**

-Vector: We digested the pGFP plasmid with the EcoRI RE and purified the

fragment (9.2kb).

-Fragment: We performed a PCR on pUAS-iab8-M3-mipep-GFP with the F

UAS_200/R UAS-deltaATG a primers, and we purified the amplified fragment

(200bp).

Gibson Assembly reaction was performed with the vector and the fragment

followed by a transformation in DH5α thermo-competent cells.

Candidate colonies were screened by digestion using the BamHI RE.

**pUAS-200-GFP-Green-F**

-Vector: We digested the pUAST-attB plasmid with the EcoRI RE and purified the

fragment (8.5kb).

-Fragments:

1-We performed a PCR on pUAS-iab8-M3-mipep-GFP with the F UAS_200/ R

G_2FS1 a primers, and we purified the amplified fragment (200bp).

191

2-We performed a PCR on pUAS-iab8-M3-mipep-GFP with the F 2_GFSN/ R

UAS_gfp primers, and we purified the amplified fragment (750bp).

Gibson Assembly reaction was performed with the vector and the fragments

followed by a transformation in DH5α thermo-competent cells.

Candidate colonies were screened by digestion using the BamHI RE.

**pUAS-200-GFP-Blue-F**

-Vector: We digested the pUAST-attB plasmid with the EcoRI RE and purified the

fragment (8.5kb).

-Fragments:

1-We performed a PCR on pUAS-iab8-M3-mipep-GFP with the F UAS_200/ R

G_2FS2 a primers, and we purified the amplified fragment (200bp).

2-We performed a PCR on pUAS-iab8-M3-mipep-GFP with the F 2_GFSN/ R

UAS_gfp primers, and we purified the amplified fragment (750bp).

Gibson Assembly reaction was performed with the vector and the fragments

followed by a transformation in DH5α thermo-competent cells.

Candidate colonies were screened by digestion using the BamHI RE.

**pUAS-200-GFP-Red-F**

-Vector: We digested the pUAST-attB plasmid with the EcoRI RE and purified the

fragment (8.5kb).

-Fragments:

1-We performed a PCR on pUAS-iab8-M3-mipep-GFP with the R G_2FS3 / R

G_2FS2 a primers, and we purified the amplified fragment (200bp).

2-We performed a PCR on pUAS-iab8-M3-mipep-GFP with the F 2_GFSN/ R

UAS_gfp primers, and we purified the amplified fragment (750bp).

Gibson Assembly reaction was performed with the vector and the fragments

followed by a transformation in DH5α thermo-competent cells.

Candidate colonies were screened by digestion using the BamHI RE.

**pUAS-ABC-GFP**

-Vector: We digested the pGFP plasmid with the EcoRI RE and purified the

fragment (9.2kb).

192

-Fragment: We performed a PCR on pUAS-iab8-M3-mipep-GFP with the F

UAS_ex1-i8/R GFP_exon1A primers, and we purified the amplified fragment

(785bp).

Gibson Assembly reaction was performed with the vector and the fragment

followed by a transformation in DH5α thermo-competent cells.

Candidate colonies were screened by digestion using the BamHI RE.

**pUAS-A-GFP**

-Vector: We digested the pGFP plasmid with the EcoRI RE and purified the

fragment (9.2kb).

-Fragment: We performed a PCR on pUAS-iab8-M3-mipep-GFP with the F

UAS_ex1-i8/R GFP_ex1A a primers, and we purified the amplified fragment

(219bp).

Gibson Assembly reaction was performed with the vector and the fragment

followed by a transformation in DH5α thermo-competent cells.

Candidate colonies were screened by digestion using the BamHI RE.

**pUAS-B-GFP**

-Vector: We digested the pGFP plasmid with the EcoRI RE and purified the

fragment (9.2kb).

-Fragment: We performed a PCR on pUAS-iab8-M3-mipep-GFP with the F

UAS_ex1B/R GFP_ex1B a primers, and we purified the amplified fragment

(402bp).

Gibson Assembly reaction was performed with the vector and the fragment

followed by a transformation in DH5α thermo-competent cells.

Candidate colonies were screened by digestion using the BamHI RE.

**pUAS-C-GFP**

-Vector: We digested the pGFP plasmid with the EcoRI RE and purified the

fragment (9.2kb).

-Fragment: We performed a PCR on pUAS-iab8-M3-mipep-GFP with the F

UAS_ex1C/R GFP_exon1 a primers, and we purified the amplified fragment

(164bp).

Gibson Assembly reaction was performed with the vector and the fragment

followed by a transformation in DH5α thermo-competent cells.

Candidate colonies were screened by digestion using the BamHI RE.

**pUAS-AB-GFP**

-Vector: We digested the pGFP plasmid with the EcoRI RE and purified the

fragment (9.2kb).

-Fragment: We performed a PCR on pUAS-iab8-M3-mipep-GFP with the F

UAS_ex1-i8/R GFP_ex1B a primers, and we purified the amplified fragment

(621bp).

Gibson Assembly reaction was performed with the vector and the fragment

followed by a transformation in DH5α thermo-competent cells.

Candidate colonies were screened by digestion using the BamHI RE.

**pUAS-BC-GFP**

-Vector: We digested the pGFP plasmid with the EcoRI RE and purified the

fragment (9.2kb).

-Fragment: We performed a PCR on pUAS-iab8-M3-mipep-GFP with the F

UAS_ex1B /R GFP_exon1 A primers, and we purified the amplified fragment

(566bp).

Gibson Assembly reaction was performed with the vector and the fragment

followed by a transformation in DH5α thermo-competent cells.

Candidate colonies were screened by digestion using the BamHI RE.

**pUAS-AC-GFP**

-Vector: We digested the pGFP plasmid with the EcoRI RE and purified the

fragment (9.2kb).

-Fragments:

1-We performed a PCR on pUAS-iab8-M3-mipep-GFP with the F UAS_ex1-i8/ R

C_ex1A a primers, and we purified the amplified fragment (219bp).

2-We performed a PCR on pUAS-iab8-M3-mipep-GFP with the F A_ex1C/R

GFP_exon1 primers, and we purified the amplified fragment (164bp).

Gibson Assembly reaction was performed with the vector and the fragments

followed by a transformation in DH5α thermo-competent cells.

Candidate colonies were screened by digestion using the BamHI RE.

**pUAS-DE-GFP**

-Vector: We digested the pGFP plasmid with the EcoRI RE and purified the

fragment (9.2kb).

-Fragment: We performed a PCR on pUAS-iab8-M3-mipep-GFP with the F

UAS_ex2/R GFP_262B a primers, and we purified the amplified fragment

(262bp).

Gibson Assembly reaction was performed with the vector and the fragment

followed by a transformation in DH5α thermo-competent cells.

Candidate colonies were screened by digestion using the BamHI RE.

**pUAS-D-GFP**

-Vector: We digested the pGFP plasmid with the EcoRI RE and purified the

fragment (9.2kb).

-Fragment: We performed a PCR on pUAS-iab8-M3-mipep-GFP with the F

UAS_ex2/R gfp_262A a primers, and we purified the amplified fragment (155bp).

Gibson Assembly reaction was performed with the vector and the fragment

followed by a transformation in DH5α thermo-competent cells.

Candidate colonies were screened by digestion using the BamHI RE.

**pUAS-E-GFP**

-Vector: We digested the pGFP plasmid with the EcoRI RE and purified the

fragment (9.2kb).

-Fragment: We performed a PCR on pUAS-iab8-M3-mipep-GFP with the F

UAS_262B/R GFP_262B a primers, and we purified the amplified fragment

(107bp).

Gibson Assembly reaction was performed with the vector and the fragment

followed by a transformation in DH5α thermo-competent cells.

Candidate colonies were screened by digestion using the BamHI RE.

pUAS-B-small-GFP-Green-F

-Vector: We digested the pUAST-attB plasmid with the EcoRI RE and purified the

fragment (8.5kb).

-Fragments:

1-We performed a PCR on pUAS-iab8-M3-mipep-GFP with the F UAS_ex1B/R

1soloF1 a primers, and we purified the amplified fragment (173bp).

2-We performed a PCR on pUAS-iab8-M3-mipep-GFP with the F 2_GFSN/R

UAS_gfp primers, and we purified the amplified fragment (714bp).

195

Gibson Assembly reaction was performed with the vector and the fragments

followed by a transformation in DH5α thermo-competent cells.

Candidate colonies were screened by digestion using the BamHI RE.

**pUAS-B-small-GFP-Blue-F**

-Vector: We digested the pUAST-attB plasmid with the EcoRI RE and purified the

fragment (8.5kb).

-Fragments:

1-We performed a PCR on pUAS-iab8-M3-mipep-GFP with the F UAS_ex1B/R

1soloF2 a primers, and we purified the amplified fragment (173bp).

2-We performed a PCR on pUAS-iab8-M3-mipep-GFP with the F 2_GFSN/ R

UAS_gfp primers, and we purified the amplified fragment (714bp).

Gibson Assembly reaction was performed with the vector and the fragments

followed by a transformation in DH5α thermo-competent cells.

Candidate colonies were screened by digestion using the BamHI RE.

**pUAS-B-small-GFP-Red-F**

-Vector: We digested the pUAST-attB plasmid with the EcoRI RE and purified the

fragment (8.5kb).

-Fragments:

1-We performed a PCR on pUAS-iab8-M3-mipep-GFP with the F UAS_ex1B/R

1soloF3 a primers, and we purified the amplified fragment (173bp).

2-We performed a PCR on pUAS-iab8-M3-mipep-GFP with the F 2_GFSN/ R

UAS_gfp primers, and we purified the amplified fragment (714bp).

Gibson Assembly reaction was performed with the vector and the fragments

followed by a transformation in DH5α thermo-competent cells.

Candidate colonies were screened by digestion using the BamHI RE.

**pUAS-B-big-GFP-Red-F/ pUAS-B-GFP-Red-F**

-Vector: We digested the pUAST-attB plasmid with the EcoRI RE and purified the

fragment (8.5kb).

-Fragments:

1-We performed a PCR on pUAS-iab8-M3-mipep-GFP with the F UAS_1trpl1/ R

1trplF1 a primers, and we purified the amplified fragment (313bp).

196

2-We performed a PCR on pUAS-iab8-M3-mipep-GFP with the F 2_GFSN/ R

UAS_gfp primers, and we purified the amplified fragment (714bp).

Gibson Assembly reaction was performed with the vector and the fragments

followed by a transformation in DH5α thermo-competent cells.

Candidate colonies were screened by digestion using the BamHI RE.

**pUAS-B-big-GFP-Green-F/ pUAS-B-GFP-Green-F**

-Vector: We digested the pUAST-attB plasmid with the EcoRI RE and purified the

fragment (8.5kb).

-Fragments:

1-We performed a PCR on pUAS-iab8-M3-mipep-GFP with the F UAS_1trpl1/ R

1trplF2 a primers, and we purified the amplified fragment (313bp).

2-We performed a PCR on pUAS-iab8-M3-mipep-GFP with the F 2_GFSN/ R

UAS_gfp primers, and we purified the amplified fragment (714bp).

Gibson Assembly reaction was performed with the vector and the fragments

followed by a transformation in DH5α thermo-competent cells.

Candidate colonies were screened by digestion using the BamHI RE.

**pUAS-B-big-GFP-Blue-F/ pUAS-B-GFP-Blue-F**

-Vector: We digested the pUAST-attB plasmid with the EcoRI RE and purified the

fragment (8.5kb).

-Fragments:

1-We performed a PCR on pUAS-iab8-M3-mipep-GFP with the F UAS_1trpl1/ R

1trplF3 a primers, and we purified the amplified fragment (313bp).

2-We performed a PCR on pUAS-iab8-M3-mipep-GFP with the F 2_GFSN/ R

UAS_gfp primers, and we purified the amplified fragment (714bp).

Gibson Assembly reaction was performed with the vector and the fragments

followed by a transformation in DH5α thermo-competent cells.

Candidate colonies were screened by digestion using the BamHI RE.

**pUAS-D- GFP-Red-F**

-Vector: We digested the pUAST-attB plasmid with the EcoRI RE and purified the

fragment (8.5kb).

-Fragments:

-We performed a PCR on pUAS-iab8-M3-mipep-GFP with the F UAS_ex2/R

GFP_262F1 a primers, and we purified the amplified fragment (155bp).

2-We performed a PCR on pUAS-iab8-M3-mipep-GFP with the F 2_GFSN/ R

UAS_gfp primers, and we purified the amplified fragment (714bp).

Gibson Assembly reaction was performed with the vector and the fragments

followed by a transformation in DH5α thermo-competent cells.

Candidate colonies were screened by digestion using the BamHI RE.

**pUAS-D- GFP-Green-F**

-Vector: We digested the pUAST-attB plasmid with the EcoRI RE and purified the

fragment (8.5kb).

-Fragments:

1-We performed a PCR on pUAS-iab8-M3-mipep-GFP with the F UAS_ex2 R

GFP_262F2 a primers, and we purified the amplified fragment (155bp).

2-We performed a PCR on pUAS-iab8-M3-mipep-GFP with the F 2_GFSN/R

UAS_gfp primers, and we purified the amplified fragment (714bp).

Gibson Assembly reaction was performed with the vector and the fragments

followed by a transformation in DH5α thermo-competent cells.

Candidate colonies were screened by digestion using the BamHI RE.

**pUAS-D- GFP-Blue-F**

-Vector: We digested the pUAST-attB plasmid with the EcoRI RE and purified the

fragment (8.5kb).

-Fragments:

1-We performed a PCR on pUAS-iab8-M3-mipep-GFP with the F UAS_ex2/R

GFP_262F3 a primers, and we purified the amplified fragment (155bp).

2-We performed a PCR on pUAS-iab8-M3-mipep-GFP with the F 2_GFSN/ R

UAS_gfp primers, and we purified the amplified fragment (714bp).

Gibson Assembly reaction was performed with the vector and the fragments

followed by a transformation in DH5α thermo-competent cells.

Candidate colonies were screened by digestion using the BamHI RE.

**pUAS-ΔATG-B-small-Blue-F**

-Vector: We digested the pUAST-attB plasmid with the EcoRI RE and purified the

fragment (8.5kb).

-Fragments:

1-We performed a PCR on pUAS-iab8-M3-mipep-GFP with the F B-delatATG/R

1soloF2 a primers, and we purified the amplified fragment (172bp).

2-We performed a PCR on pUAS-iab8-M3-mipep-GFP with the F 2_GFSN/ R

UAS_gfp primers, and we purified the amplified fragment (714bp).

Gibson Assembly reaction was performed with the vector and the fragments

followed by a transformation in DH5α thermo-competent cells.

Candidate colonies were screened by digestion using the BamHI RE.

**pUAS-ΔATG-B-small-Red-F**

-Vector: We digested the pUAST-attB plasmid with the EcoRI RE and purified the

fragment (8.5kb).

-Fragments:

1-We performed a PCR on pUAS-iab8-M3-mipep-GFP with the F B-delatATG/R

1soloF3 a primers, and we purified the amplified fragment (171bp).

2-We performed a PCR on pUAS-iab8-M3-mipep-GFP with the F 2_GFSN/ R

UAS_gfp primers, and we purified the amplified fragment (714bp).

Gibson Assembly reaction was performed with the vector and the fragments

followed by a transformation in DH5α thermo-competent cells.

Candidate colonies were screened by digestion using the BamHI RE.

**pUAS-ΔATG-B -Red-F**

-Vector: We digested the pUAST-attB plasmid with the EcoRI RE and purified the

fragment (8.5kb).

-Fragments:

1-We performed a PCR on pUAS-iab8-M3-mipep-GFP with the F B-delatATG/ R

1trplF1 a primers, and we purified the amplified fragment (339bp).

2-We performed a PCR on pUAS-iab8-M3-mipep-GFP with the F 2_GFSN/ R

UAS_gfp primers, and we purified the amplified fragment (714bp).

Gibson Assembly reaction was performed with the vector and the fragments

followed by a transformation in DH5α thermo-competent cells.

Candidate colonies were screened by digestion using the BamHI RE.

**pUAS-ΔATG-B -Green-F**

-Vector: We digested the pUAST-attB plasmid with the EcoRI RE and purified the

fragment (8.5kb).

-Fragments:

1-We performed a PCR on pUAS-iab8-M3-mipep-GFP with the F B-delatATG/ R

1trplF2 a primers, and we purified the amplified fragment (339bp).

2-We performed a PCR on pUAS-iab8-M3-mipep-GFP with the F 2_GFSN/ R

UAS_gfp primers, and we purified the amplified fragment (714bp).

Gibson Assembly reaction was performed with the vector and the fragments

followed by a transformation in DH5α thermo-competent cells.

Candidate colonies were screened by digestion using the BamHI RE.

**pUAS-ΔATG-B -Blue-F**

-Vector: We digested the pUAST-attB plasmid with the EcoRI RE and purified the

fragment (8.5kb).

-Fragments:

1-We performed a PCR on pUAS-iab8-M3-mipep-GFP with the F B-delatATG/ R

1trplF3 a primers, and we purified the amplified fragment (339bp).

2-We performed a PCR on pUAS-iab8-M3-mipep-GFP with the F 2_GFSN/ R

UAS_gfp primers, and we purified the amplified fragment (714bp).

Gibson Assembly reaction was performed with the vector and the fragments

followed by a transformation in DH5α thermo-competent cells.

Candidate colonies were screened by digestion using the BamHI RE.

**p UASexon1&2-ΔATG**

-Vector: We digested the pUAST-attB plasmid with the EcoRI RE and purified the

fragment (8.5kb).

-Fragment: The fragment was a gBlock fragment “fragment 29” ordered at IDT.

(1247bp). This fragment is a mutagenized version of the exon1&2 in which all

ATG sequences are mutated in GCC codon.

Gibson Assembly reaction was performed with the vector and the fragment

followed by a transformation in DH5α thermo-competent cells.

Candidate colonies were screened by digestion using the BamHI RE.

**pUAS-AB-small-GFP**

-Vector: We digested the pUAST-attB plasmid with the EcoRI RE and purified the

fragment (8.5kb).

-Fragments:

1-We performed a PCR on pUAS-iab8-M3-mipep-GFP with the F UAS_ex1-i8/R

1soloF3 a primers, and we purified the amplified fragment (393bp).

2-We performed a PCR on pUAS-iab8-M3-mipep-GFP with the F 2_GFSN/ R

UAS_gfp primers, and we purified the amplified fragment (714bp).

Gibson Assembly reaction was performed with the vector and the fragments

followed by a transformation in DH5α thermo-competent cells.

Candidate colonies were screened by digestion using the BamHI RE.

**pNProsyattPinPminit**

-Vector: We digested the pMiniT plasmid with the EcoRI RE and purified the

vector fragment (2.5kb).

-Fragments:

1- We digested the pSKattBflFabFRYTry plasmid with the HindIII RE and purified

the rosy gene fragment (7.3kb).

2-We performed a PCR on ry506 DNA fly prep with the NP F Prom F/NP R prom R

primers, and we purified the amplified fragment (616bp).

203

3- We performed a PCR on ry506 DNA fly prep with the NP F end F /NP R prom R

primers, and we purified the amplified fragment (637bp).

Gibson Assembly reaction was performed with the vector and the fragments

followed by a transformation in DH5α thermo-competent cells.

Candidate colonies were screened by digestion using the EcoRI RE.

**pLanding**

-Vector: We digested the pMiniT plasmid with the EcoRI RE and purified the

vector fragment (2.5kb).

-Fragment: We ordered the gBlock NPattP70 from Eurofins Genomics GmbH. We

performed a PCR using the F Pminit-ins F/ R pminit-ins R primer on the

NPattP70 fragment and purified the amplified fragment (641bp).

Gibson Assembly reaction was performed with the vector and the fragment

followed by a transformation in DH5α thermo-competent cells.

Candidate colonies were screened by digestion using the EcoRI RE.

**pexon1-ORF1-GFP**

-Vector: We digested the planding plasmid with the EcoRI RE and purified the

vector fragment (3.1kb).

-Fragment: We performed a PCR on pUAS-ORF1-GFP with the F DON-ex1 / R

DON-ex1 primers, and we purified the amplified fragment (1514bp).

Gibson Assembly reaction was performed with the vector and the fragment

followed by a transformation in DH5α thermo-competent cells.

Candidate colonies were screened by digestion using the SmaI RE.

**pexon1-ORF2-GFP**

-Vector: We digested the planding plasmid with the EcoRI RE and purified the

vector fragment (3.1kb).

-Fragment: We performed a PCR on pUAS-ORF2-GFP with the F DON-ex1 / R

DON-ex1 primers, and we purified the amplified fragment (1514bp).

Gibson Assembly reaction was performed with the vector and the fragment

followed by a transformation in DH5α thermo-competent cells.

Candidate colonies were screened by digestion using the SmaI RE.

**pExon1-ΔORF1&2**

-Vector: We digested the planding plasmid with the EcoRI RE and purified the

vector fragment (3.1kb).

-Fragment: We performed a PCR on pUAS-ΔORF1&2 with the F DON-ex1 / R

DON-ex1 primers, and we purified the amplified fragment (448bp).

Gibson Assembly reaction was performed with the vector and the fragment

followed by a transformation in DH5α thermo-competent cells.

Candidate colonies were screened by digestion using the SmaI RE.

**pExon1-rescue**

-Vector: We digested the planding plasmid with the EcoRI RE and purified the

vector fragment (3.1kb).

-Fragment: We performed a PCR on pUAS-iab8-M3-mipep-GFP with the F DONex1

/ R DON-ex1 primers, and we purified the amplified fragment (785bp).

Gibson Assembly reaction was performed with the vector and the fragment

followed by a transformation in DH5α thermo-competent cells.

Candidate colonies were screened by digestion using the SmaI RE.

**pExon1-Gal4**

-Vector: We digested the planding plasmid with the BstBI RE and purified the

vector fragment (3.6kb).

-Fragment: We performed a PCR on pActin-Gal4 with the F BstBI-Gal4 F / R

BstBI-Gal4 R primers, and we purified the amplified fragment (2700bp).

Gibson Assembly reaction was performed with the vector and the fragment

followed by a transformation in DH5α thermo-competent cells.

Candidate colonies were screened by digestion using the SmaI RE.

**Primer list:**

• F UAS_ex1-i8

ACTCTGAATAGGGAATTGGGGGCTTATTATTTTTTGGACACTTTGCCATCAGGTCG

• R MSAEX1_ex2-i8 CCGCGCACTTCCTGGGCAACTTCCTTCGTC

• F EX2-I8_msaex1 GTTGCCCAGGAAGTGCGCGGTACCGGCTTTTAAC

• R UAS-ex8 CGGCCGCAGATCTGTTAACGCGAAAAGAGGCATTGTGAGTGG

• F UAS_exon3

ACTCTGAATAGGGAATTGGGGGCTTTTTGATTACACATCGACCCCTGG

• R EX3_ex1-i8 TGTAATCAAACGGAGACATAATAAGTAAGGAG

• F Ex1_ex3 TATGTCTCCGTTTGATTACACATCGACCCCTGG

• F UAS_ex2 ACTCTGAATAGGGAATTGGGGGCTTCCCCGAAAATGCCCAACA

• F exon8 5’ CCTGTCGCTCGAGAGATTAC

• F UAS_200bp ACTCTGAATAGGGAATTGGGGGCTTGACGTCGTCTGTTGTGAAG

• R EX3-delta200 TGTAATCAAAGCAGGTGAGTGGCGAGCAGAGCAGCATG

• F DELTA200_ex3 ACTCACCTGCTTTGATTACACATCGACCCCTGG

• F UAS_pGFP

ACTCTGAATAGGGAATTGGGAATTCGGCTTCCAAACATGGTGAGCAAGGGCGAG

• R UAS_gfp CGGCCGCAGATCTGTTAACGTTACTTGTACAGCTCGTCCATGCCG

• R GFP_200 CTCACCATGTTTGGAAGCCGCCTGGGCAACTTCCTTC

• F 2_GFSN GGAGGCAGCGCCGTGAGCAAGGGCGAGGAG

• R G_2FS1 CGCTGCCTCCCCTGGGCAACTTCCTTC

• R G_2FS2 CGCTGCCTCCCTGGGCAACTTCCTTCG

• R G_2FS3 CGCTGCCTCCTGGGCAACTTCCTTCGTC

• R gfp_262A CTCACCATGTTTGGAAGCCGTCACATCCATGATGGCTGCC

• F UAS_262B ACTCTGAATAGGGAATTGGGGGCTTAAGAGAGCGTGCTCTTGG

• R GFP_262B TCACCATGTTTGGAAGCCGGCAGGTGAGTGGCGAGCAGA

• R GFP_ex1A CTCACCATGTTTGGAAGCCGTCGGATTGGTGCACAATTGGTGC

• F UAS_ex1B

ACTCTGAATAGGGAATTGGGGGCTTCAACAAAAGCCAAAAACGCTGCAGATG

• R GFP_ex1B CTCACCATGTTTGGAAGCCGTTAATTGAAGTCACGCCCCGC

• F UAS_ex1C ACTCTGAATAGGGAATTGGGGGCTTAGTGATTTGTGGCCTCCGCTTC

• R GFP_exon1 CTCACCATGTTTGGAAGCCGCGGAGACATAATAAGTAAGGAGC

• R C_ex1A ACAAATCACTTCGGATTGGTGCACAATTGGTGC

• F A_ex1C ACCAATCCGAAGTGATTTGTGGCCTCCGCTTC

• R GFP_262F1 CGCTGCCTCCCACATCCATGATGGCTGCC

• R GFP_262F2 CGCTGCCTCCACATCCATGATGGCTGCC

• R GFP_262F3 GCTGCCTCCCATCCATGATGGCTGCC

• R 1soloF1 CGCTGCCTCCCAAAGTGCTTGTTCTTTGTC

• R 1soloF2 CGCTGCCTCCAAAGTGCTTGTTCTTTGTC

• R 1soloF3 CGCTGCCTCCAAGTGCTTGTTCTTTGTC

• F UAS_1trpl1 ACTCTGAATAGGGAATTGGGGGCTTGGGAACCCAAAAGTATACGAG

• R 1trplF1 CGCTGCCTCCCAGCTGCCAAACCGCAAGGCGGAAATG

• R 1trplF2 CGCTGCCTCCAGCTGCCAAACCGCAAGGCGGAAATG

• R 1trplF3 CGCTGCCTCCGCTGCCAAACCGCAAGGCGGAAATG

• F B-delatATG :

ACTCTGAATAGGGAATTGGGGGCTTCAACAAAAGCCAAAAACGCTGCAGGCCGGAACCCAAAAGTATACGAG

• NP F Prom F TGTATCGCTCGAGGGATCCGGATTCAGAGGCCATTGGGGGTTGAAG

• NP R prom R

GGGAGATTAAACATAGTTAAAGCTCTACGCCCCCAACTGAGAGAACTCAAAGGTTACCCCAGTTGGGGCACTACGGAAATGAGAATGAGAATGCCGAGCG

• NP F end F

ATATAAGCTCAATCAAAAGACTACGCCCCCAACTGAGAGAACTCAAAGGTTACCCCAGTTGGGGCACTACGGTGTAGCAGTAAACCCAATTTG

• NP R end R GCACATGCGGCCGCCTCGAGATATATTCATTCGAGCGGGC

• F Pminit-ins F GTGTATCGCTCGAGGGATCC

• R pminit-ins R CGCACATGCGGCCGCCTCGA

• F DON-ex1 TGTCAAAGTGAACAGCAAATATTATTTTTTGGACACTTTGCCATCAGGTCG

• R DON-ex1 TCTAGCTTATTTCTACTTACCGGAGACATAATAAGTAAGGAGC

• F BstBI-Gal4 F CTGGAGTTCCTGGAGTCGCATTCCAAACATGAAGCTACTGTCTTCTATCG

• R BstBI-Gal4 R CGAACGCGCGCGGATGCGGAGTTGAAGTGAACTTGCGGGG

• HHR2 GGTGGGCCGTTGTGCTGAAA

• Rcheck 1 GATGGAGACTCACCTGATGC

• HHL2 II GGATCCACGAGATCTTGGAGCGAGGACTTA

**Gene fragments list:**

G-blocks were synthesized by Integrated DNA Technologies Inc. (Coralville, USA) or for those cloned into pEX-A2, by Eurofins ScientificSE (Luxembourg).

***gBlock Mix7:***

ACTCTGAATAGGGAATTGGGGGCTTATTATTTTTTGGACACTTTGCCATCAGGTCGACGTCTTTGATTA

CACATCGACCCCTGGAGCGAGGACTTAACCCGACGGCAGCTGCATCAGTTTCAGTTCTCCGAGGGACAT

GTAGATGGATACTCTGGGCCCTCATCTGCGTCTTCGCGTGTGTTTGTGTGTTTTTATAACATTTGTGGA

TTACGCCTATAAAAACTACACTTCACCGCGAAGCCAGCGAAGTGGGCACCAGCTACAACAAAGGACAAT

AATTATTCTGCAAAAATGATACAACCTGTCGCTCGAGAGATTAC

***gBlock fragment 29***

ATTATTTTTTGGACACTTTGCCATCAGGTCGACGTCGCGTCGCCCGGAGCTCAAACGACGATTACTTGC

TGCCACTCGTCCTGCTCCCCGTTCGGTGGGCATTGTCCTGCGGAGCTCCTGGAGTTCCTGGAGTCGCATT

CGAAGTCTGACTGTGGGTCCCGGAGCCACGGCCTTTGTCTTCGGTGGCGGCGGCGTTGCACCAATTGTG

CACCAATCCGACAACAAAAGCCAAAAACGCTGCAGGCCGGAACCCAAAAGTATACGAGAAGTCTACTCA

AAGTATATAAAGAAAGCCAAAGAAGTGGCTGTAAATCAAAAGGAGCCGGTTCGGCATCGTTTGTTGCC

AAAAATACCAAAATTAATTATAATAGCCACAAAGAACAAGCACTTTGACCCCGCGTCCGCATCCGCATT

CGAATCCGCATCCGCGCGCGTTCGGTTGGCCAATTGTGAGCCTGGATTACAAATTCGCGTGTGCGACTT

GAGAAAAATATTGGGGGCCAAAAACGCCGAGAAAAGCCTATTTACCAGCATTTCCGCCTTGCGGTTTGG

182

CAGCTGAAGGGACAGTGTTGCAGCTTTTGGACCGGCTCCCTTGAGTTTGCGGGGCGTGACTTCAATTAA

AGTGATTTGTGGCCTCCGCTTCTGACACACGAGACGTAAATAGCGGCCCTGAATTGCCGTGCTGCTAAA

ATACGTATACTAGAGCATAGTCCTCGGCCCACCGGAGGAACGGAAACCGCCTCAAAGTTTGCTAATTGA

AGAGCTCCTTACTTATTGCCTCTCCGCCCCGAAAGCCCCCAACAAAGCCCCGTCGGCGGAATCGAAAGCC

TCGGCAATTCGGATTCCCGGCGAAGAGGCAGTGGATTTAGCCGAGCCCCGCCGCCGCCGAGCTGAAAGC

CAAGAAGAAGAGGCGAGACAGCGGCAGCCATCGCCGGCCTGAAAGAGAGCGTGCTCTTGGCCTCTCTCG

CTCGCGCTCGTGCCTACGACAGTGCGGTATTCCACAGCGGCCGCCCTCGCTCCGCCCTGCTCTGCTCGCC

ACTCACCTGCGACGTCGTCTGTTGTGAAGTTCTTGCGACGTGAGCGACCGAAACTCGGGGCCCCGAAAA

ACTAGAGGCCCAAAAAACAGGATTGTGAGCGAGCGAGGGAGCGGAGGAATTTCGAGGGACTTTCTGCG

ACTGCGTCGTCGCCCAGACAAAAACAAAAAAAGCCCAGAAACAAAGGCCGACGGACGAAGGAAGTTGCC

CAGG

***gBlock NP attP70***

AAGCTTGTGTATCGCTCGAGGGATCCCTCGAAGCCGCGGTGCGGGTGCCAGGGCGTGCCCTTGGGCTCC

CCGGGCGCGTACTCCACCTCACCCATCATTCGGATAGCCTTAGCAGTTTTAAAACATTTCGTAAGGCCC

AATATGTGTATTTAAAAGGTGGTTTAGTTCTAGCTCTAGCTTATTTCTACTTACGAATTCATTTGCTGT

TCACTTTGACACAAGGCGCGCTTGGGTTCGTTCCGTTCGTTTCGGTTGCAGCGCAGTCGACGCAGATAC

AGATATATAGATACATAGATACAAATATACCGAGGTGGTTGTCGAGGAGATGCCGGCGAGGTTGTGAG

GCTCTGAGGATGCGAGGTGAGCATGGCACAAAAACAGAGAACTCAGAACTGAGCATCGAAAACAGAAA

ACTGAGAATCGAGCATCGAGCAACGAGCAACGATAGACCCCAGAAGGGCAAAAGTGGGAAGGAAAGAA

CGCCAACCAGAAGGTCGTAAACCCGGGCAGCGCTGCCACGATGGCAACACTTGCGAGAACGCCAAGATG

GGTGAGGTGGAGTACGCGCCCGGGGAGCCCAAGGGCACGCCCTGGCACCCGCACCGCGGCTTCGAGTCG

AGGCGGCCGCATGTGCGAAGCTT

**gBlock AluI guide**

GAAAAGCCGAGTCAAATGCCGAATGCAGAGTCTCATTACAGCACAATCAACTCAAGAAAAACTCGACAC

TTTTTTACCATTTGCACTTAAATCCTTTTTTATTCGTTATGTATACTTTTTTTGGTCCCTAACCAAAAC

AAAACCAAACTCTCTTAGTCGTGCCTCTATATTTAAAACTATCAATTTATTATAGTCAATAAATCGAAC

TGTGTTTTCAACAAACGAACAATAGGACACTTTGATTCTAAAGGAAATTTTGAAAATCTTAAGCAGAG

GGTTCTTAAGACCATTTGCCAATTCTTATAATTCTCAACTGCTCTTTCCTGATGTTGATCATTTATATA

GGTATGTTTTCCTCAATACTTCGGTGGCAAAATATCAAACAAGTTTTAGAGCTAGAAATAGCAAGTTA

AAATAAGGCTAGTCCGTTATCAACTTGAAAAAGTGGCACCGAGTCGGTGCTTTTTTT

***gBlock BsmI guide***

GAAAAGCCGAGTCAAATGCCGAATGCAGAGTCTCATTACAGCACAATCAACTCAAGAAAAACTCGACAC

TTTTTTACCATTTGCACTTAAATCCTTTTTTATTCGTTATGTATACTTTTTTTGGTCCCTAACCAAAAC

AAAACCAAACTCTCTTAGTCGTGCCTCTATATTTAAAACTATCAATTTATTATAGTCAATAAATCGAAC

TGTGTTTTCAACAAACGAACAATAGGACACTTTGATTCTAAAGGAAATTTTGAAAATCTTAAGCAGAG

GGTTCTTAAGACCATTTGCCAATTCTTATAATTCTCAACTGCTCTTTCCTGATGTTGATCATTTATATA

GGTATGTTTTCCTCAATACTTCGGGCTTGCATTCATTTTTTGGTTTTAGAGCTAGAAATAGCAAGTTAA

AATAAGGCTAGTCCGTTATCAACTTGAAAAAGTGGCACCGAGTCGGTGCTTTTTTT

***Guide 3’ in pEX-A2***

CCTGCTTTTGCTCGCTTGGATCCGAATTCCTGTGTGAAATTGTTATCCGCTCACAATTCCACACAACAT

ACGAGCCGGAAGCATAAAGTGTAAAGCCTGGGGTGCCTAATGAGTGAGCTAACTCACATTAATTGCGTT

GCGCTCACTGCCCGCTTTCCAGTCGGGAAACCTGTCGTGCCAGCTGCATTAATGAATCGGCCAACGCGC

GGGGAGAGGCGGTTTGCGTATTGGGCGCTCTTCCGCTTCCTCGCTCACTGACTCGCTGCGCTCGGTCGTT

CGGCTGCGGCGAGCGGTATCAGCTCACTCAAAGGCGGTAATACGGTTATCCACAGAATCAGGGGATAAC

GCAGGAAAGAACATGTGAGCAAAAGGCCAGCAAAAGGCCAGGAACCGTAAAAAGGCCGCGTTGCTGGC

GTTTTTCCATAGGCTCCGCCCCCCTGACGAGCATCACAAAAATCGACGCTCAAGTCAGAGGTGGCGAAA

CCCGACAGGACTATAAAGATACCAGGCGTTTCCCCCTGGAAGCTCCCTCGTGCGCTCTCCTGTTCCGACC

CTGCCGCTTACCGGATACCTGTCCGCCTTTCTCCCTTCGGGAAGCGTGGCGCTTTCTCATAGCTCACGCT

GTAGGTATCTCAGTTCGGTGTAGGTCGTTCGCTCCAAGCTGGGCTGTGTGCACGAACCCCCCGTTCAGC

CCGACCGCTGCGCCTTATCCGGTAACTATCGTCTTGAGTCCAACCCGGTAAGACACGACTTATCGCCACT

GGCAGCAGCCACTGGTAACAGGATTAGCAGAGCGAGGTATGTAGGCGGTGCTACAGAGTTCTTGAAGT

GGTGGCCTAACTACGGCTACACTAGAAGAACAGTATTTGGTATCTGCGCTCTGCTGAAGCCAGTTACCT

TCGGAAAAAGAGTTGGTAGCTCTTGATCCGGCAAACAAACCACCGCTGGTAGCGGTGGTTTTTTTGTTT

GCAAGCAGCAGATTACGCGCAGAAAAAAAGGATCTCAAGAAGATCCTTTGATCTTTTCTACGGGGTCTG

ACGCTCAGTGGAACGAAAACTCACGTTAAGGGATTTTGGTCATGAGATTATCAAAAAGGATCTTCACCT

AGATCCTTTTAAATTAAAAATGAAGTTTTAAATCAATCTAAAGTATATATGAGTAAACTTGGTCTGAC

AGTTACCAATGCTTAATCAGTGAGGCACCTATCTCAGCGATCTGTCTATTTCGTTCATCCATAGTTGCC

TGACTCCCCGTCGTGTAGATAACTACGATACGGGAGGGCTTACCATCTGGCCCCAGTGCTGCAATGATA

CCGCGACTCCCACGCTCACCGGCTCCAGATTTATCAGCAATAAACCAGCCAGCCGGAAGGGCCGAGCGC

AGAAGTGGTCCTGCAACTTTATCCGCCTCCATCCAGTCTATTAATTGTTGCCGGGAAGCTAGAGTAAGT

AGTTCGCCAGTTAATAGTTTGCGCAACGTTGTTGCCATTGCTACAGGCATCGTGGTGTCACGCTCGTCG

TTTGGTATGGCTTCATTCAGCTCCGGTTCCCAACGATCAAGGCGAGTTACATGATCCCCCATGTTGTGC

AAAAAAGCGGTTAGCTCCTTCGGTCCTCCGATCGTTGTCAGAAGTAAGTTGGCCGCAGTGTTATCACTC

ATGGTTATGGCAGCACTGCATAATTCTCTTACTGTCATGCCATCCGTAAGATGCTTTTCTGTGACTGGT

GAGTACTCAACCAAGTCATTCTGAGAATAGTGTATGCGGCGACCGAGTTGCTCTTGCCCGGCGTCAATA

CGGGATAATACCGCGCCACATAGCAGAACTTTAAAAGTGCTCATCATTGGAAAACGTTCTTCGGGGCGA

AAACTCTCAAGGATCTTACCGCTGTTGAGATCCAGTTCGATGTAACCCACTCGTGCACCCAACTGATCT

TCAGCATCTTTTACTTTCACCAGCGTTTCTGGGTGAGCAAAAACAGGAAGGCAAAATGCCGCAAAAAAG

GGAATAAGGGCGACACGGAAATGTTGAATACTCATACTCTTCCTTTTTCAATATTATTGAAGCATTTAT

CAGGGTTATTGTCTCATGAGCGGATACATATTTGAATGTATTTAGAAAAATAAACAAATAGGGGTTCC

GCGCACATTTCCCCGAAAAGTGCCACCTGACGTCTAAGAAACCATTATTATCATGACATTAACCTATAA

AAATAGGCGTATCACGAGGCCCTTTCGTCTCGCGCGTTTCGGTGATGACGGTGAAAACCTCTGACACAT

GCAGCTCCCGGAGACGGTCACAGCTTGTCTGTAAGCGGATGCCGGGAGCAGACAAGCCCGTCAGGGCGC

GTCAGCGGGTGTTGGCGGGTGTCGGGGCTGGCTTAACTATGCGGCATCAGAGCAGATTGTACTGAGAGT

GCACCAATTGGGTACCGAGCTCGCGGCCGCAAGCGAAAAGCCGAGTCAAATGCCGAATGCAGAGTCTCA

TTACAGCACAATCAACTCAAGAAAAACTCGACACTTTTTTACCATTTGCACTTAAATCCTTTTTTATTC

GTTATGTATACTTTTTTTGGTCCCTAACCAAAACAAAACCAAACTCTCTTAGTCGTGCCTCTATATTTA

AAACTATCAATTTATTATAGTCAATAAATCGAACTGTGTTTTCAACAAACGAACAATAGGACACTTTG

ATTCTAAAGGAAATTTTGAAAATCTTAAGCAGAGGGTTCTTAAGACCATTTGCCAATTCTTATAATTC

TCAACTGCTCTTTCCTGATGTTGATCATTTATATAGGTATGTTTTCCTCAATACTTCGACTGCTAAGGC

TATCCGAAGTTTTAGAGCTAGAAATAGCAAGTTAAAATAAGGCTAGTCCGTTATCAACTTGAAAAAGT

GGCACCGAGTCGGTGCTTTTTTTA

***Guide 5’ in pEX-A2***

CCTGCTTTTGCTCGCTTGGATCCGAATTCCTGTGTGAAATTGTTATCCGCTCACAATTCCACACAACAT

ACGAGCCGGAAGCATAAAGTGTAAAGCCTGGGGTGCCTAATGAGTGAGCTAACTCACATTAATTGCGTT

GCGCTCACTGCCCGCTTTCCAGTCGGGAAACCTGTCGTGCCAGCTGCATTAATGAATCGGCCAACGCGC

GGGGAGAGGCGGTTTGCGTATTGGGCGCTCTTCCGCTTCCTCGCTCACTGACTCGCTGCGCTCGGTCGTT

CGGCTGCGGCGAGCGGTATCAGCTCACTCAAAGGCGGTAATACGGTTATCCACAGAATCAGGGGATAAC

GCAGGAAAGAACATGTGAGCAAAAGGCCAGCAAAAGGCCAGGAACCGTAAAAAGGCCGCGTTGCTGGC

GTTTTTCCATAGGCTCCGCCCCCCTGACGAGCATCACAAAAATCGACGCTCAAGTCAGAGGTGGCGAAA

CCCGACAGGACTATAAAGATACCAGGCGTTTCCCCCTGGAAGCTCCCTCGTGCGCTCTCCTGTTCCGACC

CTGCCGCTTACCGGATACCTGTCCGCCTTTCTCCCTTCGGGAAGCGTGGCGCTTTCTCATAGCTCACGCT

GTAGGTATCTCAGTTCGGTGTAGGTCGTTCGCTCCAAGCTGGGCTGTGTGCACGAACCCCCCGTTCAGC

CCGACCGCTGCGCCTTATCCGGTAACTATCGTCTTGAGTCCAACCCGGTAAGACACGACTTATCGCCACT

GGCAGCAGCCACTGGTAACAGGATTAGCAGAGCGAGGTATGTAGGCGGTGCTACAGAGTTCTTGAAGT

GGTGGCCTAACTACGGCTACACTAGAAGAACAGTATTTGGTATCTGCGCTCTGCTGAAGCCAGTTACCT

TCGGAAAAAGAGTTGGTAGCTCTTGATCCGGCAAACAAACCACCGCTGGTAGCGGTGGTTTTTTTGTTT

GCAAGCAGCAGATTACGCGCAGAAAAAAAGGATCTCAAGAAGATCCTTTGATCTTTTCTACGGGGTCTG

ACGCTCAGTGGAACGAAAACTCACGTTAAGGGATTTTGGTCATGAGATTATCAAAAAGGATCTTCACCT

AGATCCTTTTAAATTAAAAATGAAGTTTTAAATCAATCTAAAGTATATATGAGTAAACTTGGTCTGAC

AGTTACCAATGCTTAATCAGTGAGGCACCTATCTCAGCGATCTGTCTATTTCGTTCATCCATAGTTGCC

TGACTCCCCGTCGTGTAGATAACTACGATACGGGAGGGCTTACCATCTGGCCCCAGTGCTGCAATGATA

CCGCGACTCCCACGCTCACCGGCTCCAGATTTATCAGCAATAAACCAGCCAGCCGGAAGGGCCGAGCGC

AGAAGTGGTCCTGCAACTTTATCCGCCTCCATCCAGTCTATTAATTGTTGCCGGGAAGCTAGAGTAAGT

AGTTCGCCAGTTAATAGTTTGCGCAACGTTGTTGCCATTGCTACAGGCATCGTGGTGTCACGCTCGTCG

TTTGGTATGGCTTCATTCAGCTCCGGTTCCCAACGATCAAGGCGAGTTACATGATCCCCCATGTTGTGC

AAAAAAGCGGTTAGCTCCTTCGGTCCTCCGATCGTTGTCAGAAGTAAGTTGGCCGCAGTGTTATCACTC

ATGGTTATGGCAGCACTGCATAATTCTCTTACTGTCATGCCATCCGTAAGATGCTTTTCTGTGACTGGT

GAGTACTCAACCAAGTCATTCTGAGAATAGTGTATGCGGCGACCGAGTTGCTCTTGCCCGGCGTCAATA

CGGGATAATACCGCGCCACATAGCAGAACTTTAAAAGTGCTCATCATTGGAAAACGTTCTTCGGGGCGA

AAACTCTCAAGGATCTTACCGCTGTTGAGATCCAGTTCGATGTAACCCACTCGTGCACCCAACTGATCT

TCAGCATCTTTTACTTTCACCAGCGTTTCTGGGTGAGCAAAAACAGGAAGGCAAAATGCCGCAAAAAAG

GGAATAAGGGCGACACGGAAATGTTGAATACTCATACTCTTCCTTTTTCAATATTATTGAAGCATTTAT

CAGGGTTATTGTCTCATGAGCGGATACATATTTGAATGTATTTAGAAAAATAAACAAATAGGGGTTCC

GCGCACATTTCCCCGAAAAGTGCCACCTGACGTCTAAGAAACCATTATTATCATGACATTAACCTATAA

AAATAGGCGTATCACGAGGCCCTTTCGTCTCGCGCGTTTCGGTGATGACGGTGAAAACCTCTGACACAT

GCAGCTCCCGGAGACGGTCACAGCTTGTCTGTAAGCGGATGCCGGGAGCAGACAAGCCCGTCAGGGCGC

GTCAGCGGGTGTTGGCGGGTGTCGGGGCTGGCTTAACTATGCGGCATCAGAGCAGATTGTACTGAGAGT

GCACCAATTGGGTACCGAGCTCGCGGCCGCAAGCGAAAAGCCGAGTCAAATGCCGAATGCAGAGTCTCA

TTACAGCACAATCAACTCAAGAAAAACTCGACACTTTTTTACCATTTGCACTTAAATCCTTTTTTATTC

GTTATGTATACTTTTTTTGGTCCCTAACCAAAACAAAACCAAACTCTCTTAGTCGTGCCTCTATATTTA

AAACTATCAATTTATTATAGTCAATAAATCGAACTGTGTTTTCAACAAACGAACAATAGGACACTTTG

ATTCTAAAGGAAATTTTGAAAATCTTAAGCAGAGGGTTCTTAAGACCATTTGCCAATTCTTATAATTC

TCAACTGCTCTTTCCTGATGTTGATCATTTATATAGGTATGTTTTCCTCAATACTTCGCTCGCAAGTGT

TGCCATCGGTTTTAGAGCTAGAAATAGCAAGTTAAAATAAGGCTAGTCCGTTATCAACTTGAAAAAGT

GGCACCGAGTCGGTGCTTTTTTTA
