# Supplementary figures and images for: Upstream open reading frames repress the translation from the iab-8 RNA
Source: PLoS Genet. 2024 Sep 23;20(9):e1011214. doi: 10.1371/journal.pgen.1011214 (PMC11463788; doi:10.1371/journal.pgen.1011214)

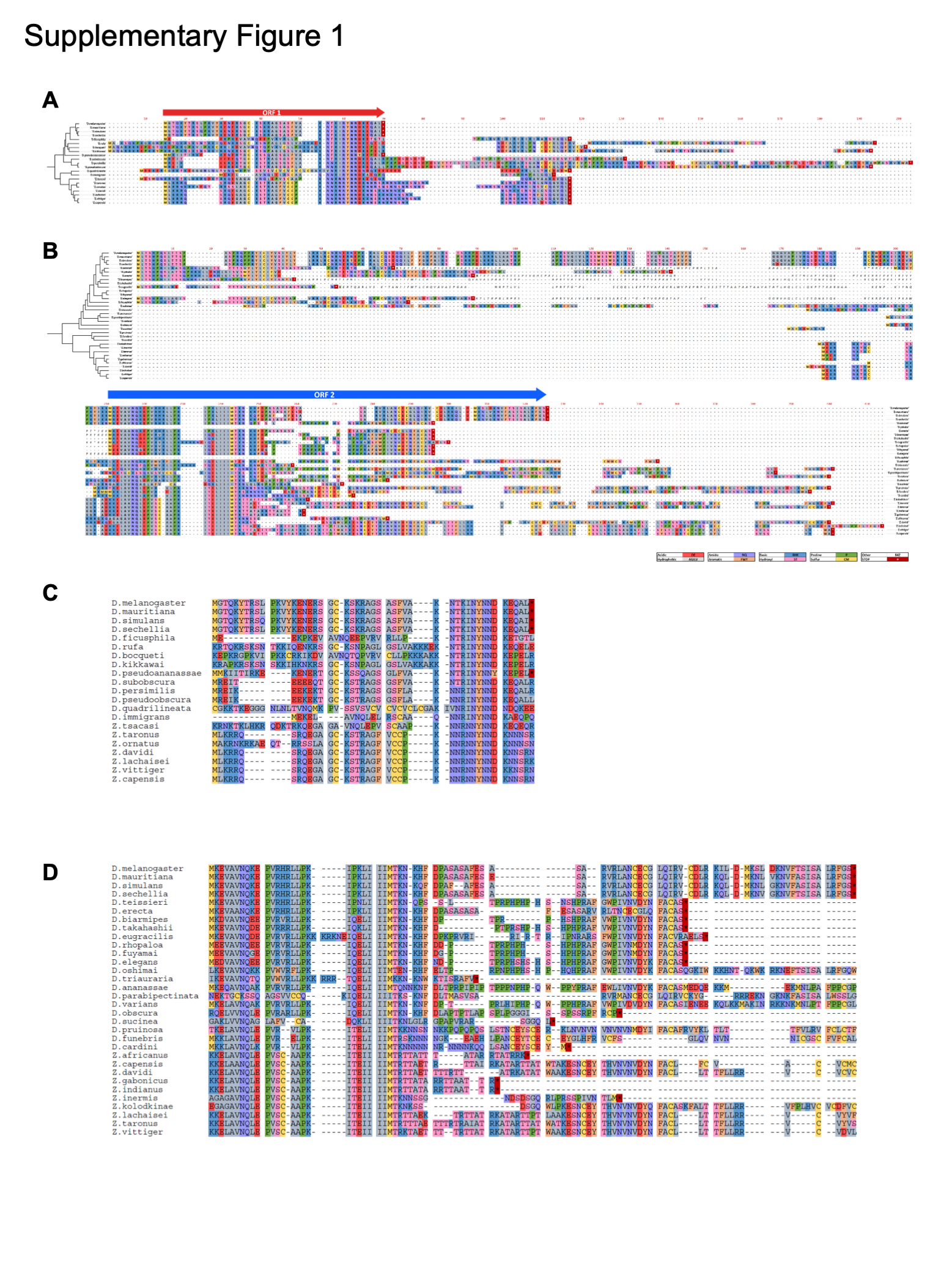

Supplement: S1 Fig — Amino acid alignment results for ORF 1 (A.), and ORF 2 (B.) across 44 Drosophilid species. The ’X’ characters in the amino acids sequences indicates that one or two additional bases have been added to the nucleotide sequence to reveal the conservation of both the beginning and the end of ORF 2 on two different reading frames in some species (teissieri, biarmipes eugracilis). Italicized characters indicate residues that are not part of any open reading frame, located after a STOP codon and before a following Methionine. C. D. are closer looks at the alignments of the main parts of ORF1 (C) and ORF2 (D). The alignments were obtained using the muscle algorithm available on the SeaView sequence analysis application. Species names and phylogeny 37 are indicated at the left of each alignment. Amino acid residues are color coded according to the legend at the bottom of the figure. (TIFF) [file pgen.1011214.s002.tiff]

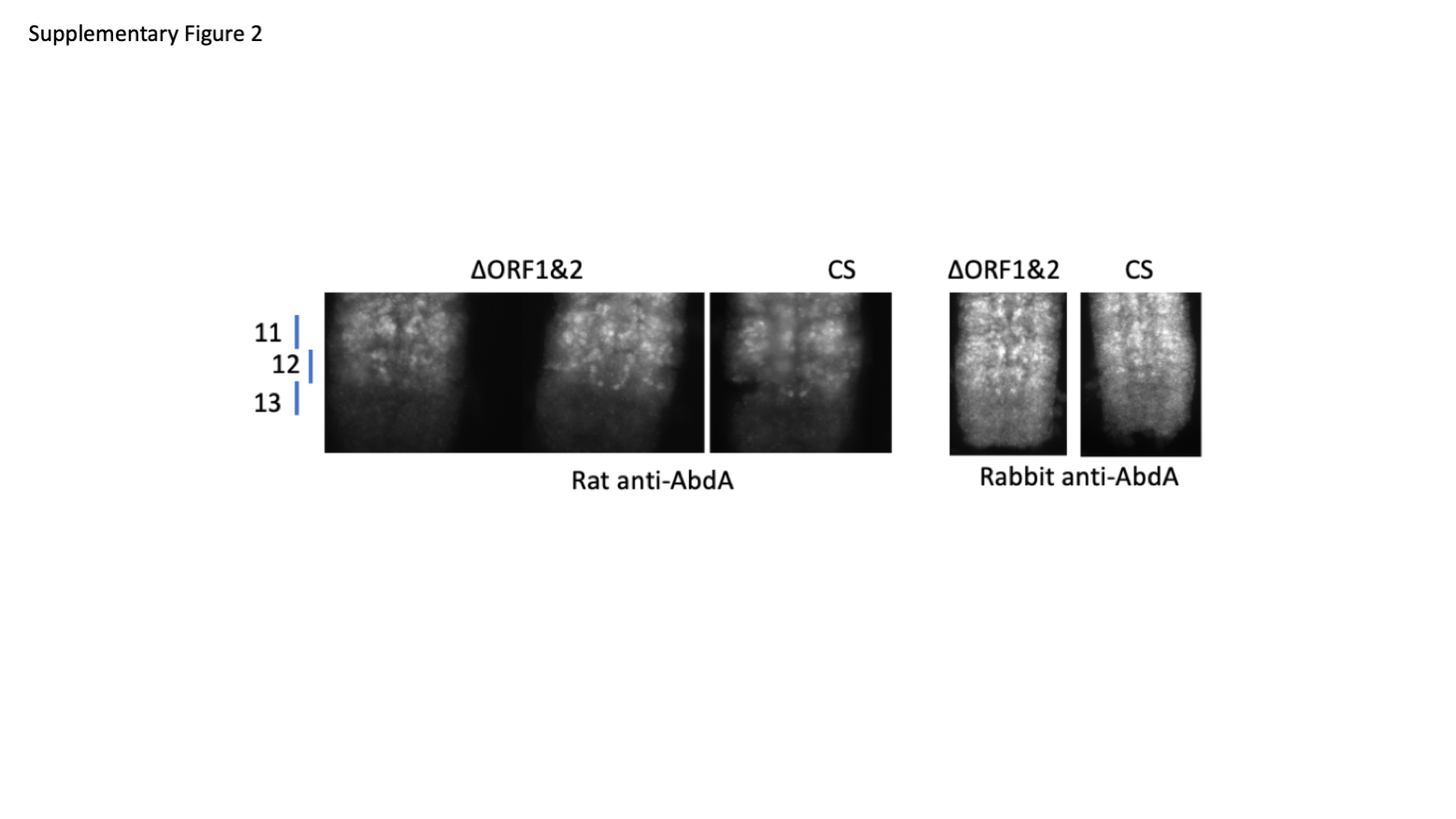

Supplement: S2 Fig — The developing nerve chords were dissected from stage 15 embryos after staining against ABD-A using either a rat anti-ABD-A antibody or a rabbit anti-ABD-A antibody. The location of parasegment 11–13 are marked on the left. (TIFF) [file pgen.1011214.s003.tiff]

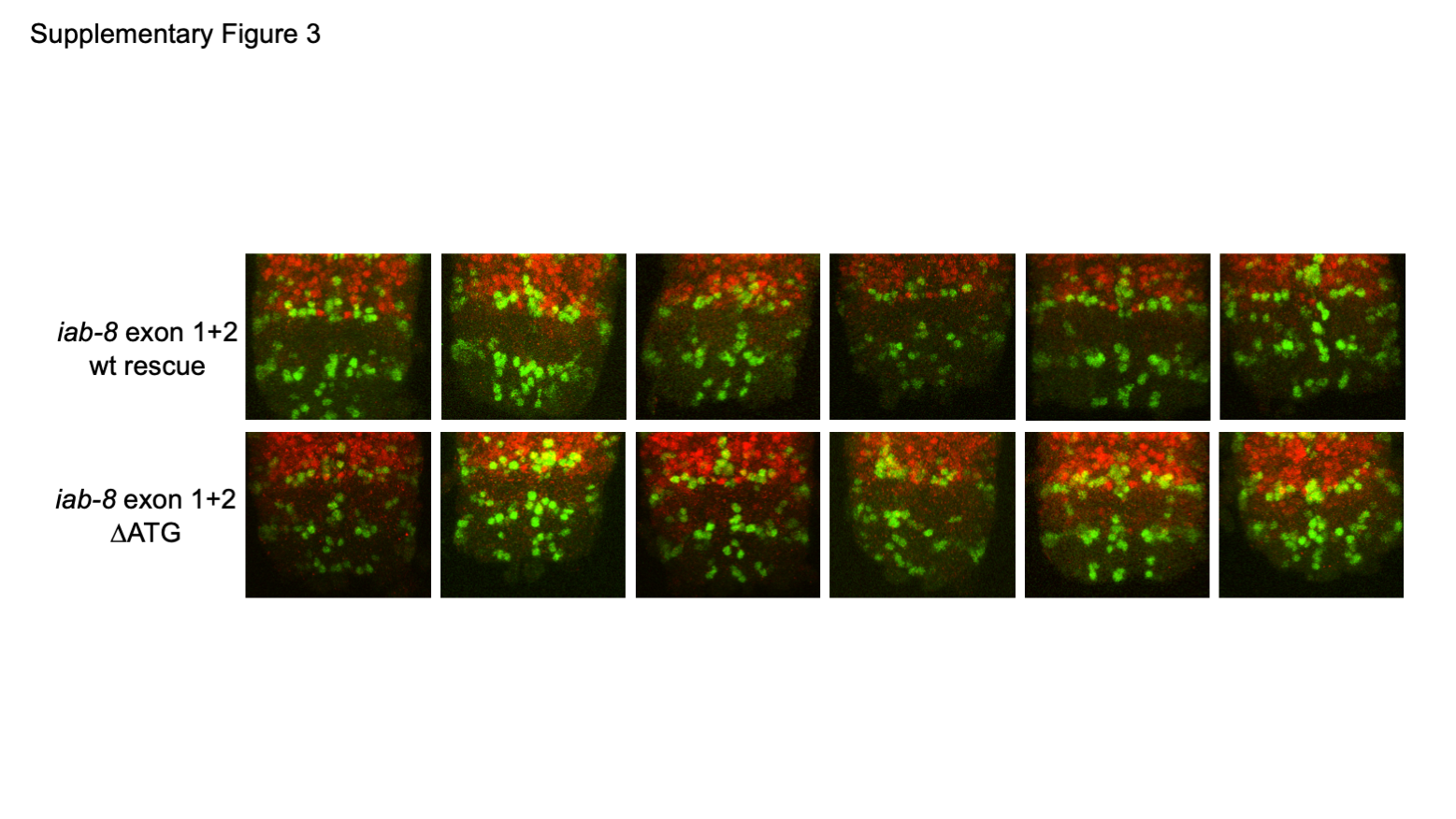

Supplement: S3 Fig — The developing nerve chords were dissected from stage 15 embryos after staining against ABD-A using the goat anti-ABD-A, DH-17 antibody (red) and engrailed as a parasegment marker (40). The top six images are of posterior nerve chords dissected from an iab-8 rescue line and the bottom six are examples dissected from an iab-8 Exon 1+2 Delta ATG line. (TIFF) [file pgen.1011214.s004.tiff]
