# Supplementary material for: Upstream open reading frames repress the translation from the iab-8 RNA
Source: PLoS Genet. 2024 Sep 23;20(9):e1011214. doi: 10.1371/journal.pgen.1011214 (PMC11463788; doi:10.1371/journal.pgen.1011214)
Supplement: Attachment — Submitted filename: Comments on Yohan Frei et al_SN-DV.docx [file pgen.1011214.s011.docx]

**Comments on Yohan Frei et al (submitted to Plos Genetics)**

**Summary**

A vast body of work recognizes the physiological importance of so-called long non coding RNAs (lncRNAs), but recent studies have revealed that these lncRNAs can actually encode for micropeptides (miPeps) in addition to microRNAs (miRNAs). This work by Frei et al demonstrate that intricate translation regulation governs the expression of miPeps, and that such regulation has an outsized impact on physiology. The study investigates the translation regulation of the male-specific abdominal (MSA) transcript in *Drosophila*, which presents as two splice isoforms, one capable of encoding a micropeptide (MSAmiP) and another splice variant (called iab-lncRNA) incapable of doing so despite bearing the MSAmiP coding region. MSAmiP is produced in the secondary cells of male accessory glands and has been shown to be an important component of male seminal fluids, important for inducing postmating responses in females. The mechanisms underlying the seeming suppression of MSAmiP translation off the iab-8 lncRNA is unknown.

The authors use an extremely thorough array of control and mutant transgenic reporters in S2-cell based assays to investigate the upstream 5’ elements of iab-8 lncRNA. Their results reveal a prominent role for upstream open reading frames (uORFs) present in exons 1 and 2 (which are unique to iab-8 lncRNA and absent in the other MSA mRNA variant) in the suppression of MSAmiP translation. They break down these exons in careful 5’ “bashing” studies to show that translation initiation off a competent AUG in exon 1 acts as the primary suppressor of MSAmiP production off the iab-8 lncRNA. They also perform bioinformatic analyses across 44 different Drosophilid species to show the conservation of this ORF across evolutionary scales. The paper concludes with in vivo evidence where mutation of the relevant exon 1 sequences in the iab-8 lncRNA now results in spurious MSAmiP production. Overall this paper does a careful analysis of the translational regulation of the MSAmiP peptide which is important for fly fertility and establishes a necessary precedent in the study of lncRNA biology. The data in the paper are compelling, and the conclusions thus warranted. Regrettably, the paper suffers from inconsistent writing and confusing discussion in some parts. Barring heavy editing required to improve the readability of the paper and some minor clarifying experiments, I am fully in support of publication of this work. I’ve detailed my editorial suggestions and a couple of experimental concerns below.

Experimental concerns:

- In Fig6E, the wing discs appear to be of different sizes. Are the larvae age-matched or is the difference in wing size a phenotype? Also, overexpression of UAS-exon1&2dATG appears to have an effect on tissue health in the accessory gland, is this tissue specific? Compartment specific overexpression of the same shows increased total wing size, or is the representative image simply from a slightly different developmental stage? Please clarify in text.

Also, the wing disc images suggest incomplete penetration of the DAPI dye - please explain why the images only show DAPI staining on the margins of wing imaginal discs, and not within the notum and pouch.

- The authors genetically manipulate accessory gland by ectopic reporter expression (Fig. 6) and by mutating the iab-8 lncRNA. However, there is very little clarity on the effects of these genetic manipulations on fertility, mating status, post mating response etc. It would be helpful to describe these phenotypes to fully appreciate the role of translation regulation of MSAmiPEP in male fertility.
- The strongest physiological relevance for this study comes from their experiments described in page 16 paragraph 2, where the endogenous exon1/2 is replaced with a mutant one “lacking uORFs”. It is unclear what the authors mean by “lacking uORFs” especially in the milieu of all the uORF mutants they use in the paper. The authors describe that these exon1/2 mutants essentially act as iab-8 lncRNA nulls and show male sterility. However, no data is presented for any of these claims? Specifically no control is shown that splicing of the normal MSAmiPep-encoding MSA mRNA is unaffected by the mutation in exons 1/2. Similarly, it is unclear if the mutations in exon1/2 have any impact on miRNA generation off of the iab-8 lncRNA template.

Editing recommendations

- As it stands the manuscript has a ‘results and discussion’ section - for the sake of readability and elaboration, as well as conforming to journal standards, it would be very useful if the authors separated the sections.
- The references to figures after description of the data is inconsistent throughout the manuscript. *e.g,* in some places, discussion of the data begins with “as see in figure xxx” (page 12, end of second paragraph), some places don’t reference a figure number (page 12, penultimate line). I would recommend consistently referencing the relevant figure for each data and discussion point throughout the manuscript to enable the reader to follow the authors’ rationale.
- Please ensure consistent labeling across figures, especially of the genotypes and fluorescent markers, particularly Figure 7.
- The images in figure 6 and S2 (among others) are of lower resolution with barely readable text. Hopefully this will be resolved for publication.
- Page 16 paragraph 2: s/spiced/spliced
- Fig 1.b - Y axis label reads ‘Fluo’ - it can be expanded to say ‘GFP fluorescence’
- Ensure that *Drosophila* is consistently italicised across the text.
- The titles of the figure legend should reflect the key finding reported in that figure instead of a brief description of the experiment performed.
- In the figure legends, please be consistent and ensure the sub figures are labelled (for example, Figure 1B is simply mentioned as ‘the bottom panel’ but A. is mentioned in the legend. Some figure legends also have the sub figure letter in bold (fig 1A in the legend) but not in others (fig. 2A in legend). This is also confusing to read since figure 3 has fragments labelled as ‘A,B…F’ but the legend makes no mention of the sub figure labels. Please correct this appropriately to make it easier for the reader to understand the study.
- DAPI is all capitals since it is an abbreviation, please correct in text and figures.
- Page 8, “mixes of plasmids were mixed” is redundant.
- In figure 3, the authors divide up exons1/2 into A-F regions, which is quite helpful in navigating this complex mRNA. However, it would serve them well to introduce this method of representing the exons1/2 prior to the data in Fig. 2.
