# Supplementary material for: Upstream open reading frames repress the translation from the iab-8 RNA
Source: PLoS Genet. 2024 Sep 23;20(9):e1011214. doi: 10.1371/journal.pgen.1011214 (PMC11463788; doi:10.1371/journal.pgen.1011214)
Supplement: Attachment — Submitted filename: Frei_response_to_reviewers..docx [file pgen.1011214.s012.docx]

Dear PloS Genetics:

Please find below our responses to the reviewer concerns regarding our manuscript currently entitled “**Upstream open reading frames repress the translation from the *iab-8* RNA”** by Frei et al. Overall, we were quite happy with the reviews, which largely seemed to center around a clarification of the text and figures. Thus, we have remade almost all of the figures and substantially modified the text in hopes of making the manuscript easier to read and less confusing. Aside from the general rewriting, the changes of note include: a modification of the title to lessen the emphasis on the regulation of the MSAmiP micropeptide, changing and adding images to examine the regulation in fly tissues and a true division of the results and discussion sections. This last change allowed us more freedom to place our findings into perspective with regards to the other uORF systems.

We have placed the specific reviewer comments below, each followed by our responses in red.

Reviewer #1: The study investigates the translational regulation of a small peptide, MSAmiP, in Drosophila melanogaster. The study reveals that the male-specific abdominal (MSA) RNA, initially classified as non-coding, actually encodes for MSAmiP, which is produced in the male accessory gland and is crucial for sperm competition. The researchers discovered that the lack of MSAmiP synthesis in the central nervous system (CNS) is due to upstream open reading frames (uORFs) present in the iab-8 lncRNA, which is transcribed from an alternate promoter. Through cell culture and transgenic analysis, the authors demonstrate that these uORFs prevent the production of protein from downstream ORFs. The findings suggest that uORFs play a role in regulating gene expression and may have evolved to prevent unwanted translation of certain transcripts, such as those resulting from alternative splicing. The study provides insights into post-transcriptional regulation's complexity and non-coding RNAs' role in gene expression control. Overall, the experiments are solid and support their conclusion. My concerns are listed below.

We would like to thank the reviewer for their time and for their appreciation of our work.

1.The illustration of different reporter constructs in Figures 2-5 is hard to understand, especially the different reading frames of start codons and GFP reporters.

Yes, in retrospect we realized the difficulty and have tried to simplify both the text and the figures. Regarding the figures, in particular, we have added an aspect to the construct diagrams, where constructs that express GFP have the GFP portion of the construct shown in green to help summarize the results at a glance. We have also tried to clean up the figures to make them less busy. The start codons are now represented as arrows and the stop codons by stop sign like icons. We thought that this would make the mental load a bit lighter. But, we also tried to change the text to highlight the main issues and lessen the need for the reader to wade through the figures to find the main points.

2. While the authors have provided a detailed dissection of uORF-mediated translation regulation for MSAmiP, similar mechanisms of regulating protein translation by switching isoforms with different numbers of uORFs have been proposed by several groups as reviewed here (DOI: 10.1016/j.tibs.2019.03.002). It is better to discuss connections with previous studies.

Thank you for this suggestion. Previously, we thought it beneficial to keep the manuscript as short as possible but appreciate this reviewer’s point. We now have a divided results and discussion sections and have added a discussion on the use of uORFs in controlling translation in other systems (with the review suggested by the referee taken into account).

3**. "But, this begs the question: were the upstream open reading frames added to the iab-8 RNA to prevent spurious abd-A expression or are they perhaps remnants of a coding gene now potentially in the process of degeneration? The limited conservation of the ORFs may support the latter." Suppose the uORFs in exons 1 and 2 are regulatory. In that case, there is no need for strong conservation for ORF sequence, and even the position of uORF start codons does not matter as long as there are uORFs with strong translatability. Therefore, I do not see why the latter scenario is more likely.**

This is a good point. We were thinking of this as one possible explanation of the lack of an overt phenotype. Of course, we know that the removal of many genes has no noticeable phenotype in the laboratory, even if they are conserved. In any case, we have modified the discussion to include the reviewer input.

**4. To further demonstrate that this is a conserved regulatory mechanism, could the authors show that the iab-8 and MSA** transcript isoforms are conserved in other Drosophila species with public data?

Much of this has already been done. We know that the *iab-8* miRNA with its placement next to *abd-A* is conserved in many examined arthropods (Pace et al. EvoDevo 2016). We also know that the target sites in the hox genes are also well conserved based on work from the Lai lab (Tyler et al 2008). In their work, the authors show that multiple sites for *mir-iab-8* (distinguishable from *mir-iab-4* transcribed from the opposite strand) are conserved in both *abd-A and Ubx*. Although these studies are primarily concerned with the miRNA and not the transcript itself, its conserved placement and target conservation is highly suggestive. Lastly, work from our lab has confirmed that the *iab-8* transcript is expressed in *D. virilis* (Gummalla 2012) with a pattern identical to that of *D. melanogaster*. As virilis is located on the *Drosophila* branch of the *Drosophila* tree (rather than *Sophophora* branch, where melanogaster is located), we believe that the mechanism could definitely be conserved.

Regarding the *MSA* transcript, most bioinformatic studies do not make a distinction between *iab-8* and *MSA* (as they share most sequences). Even in *melanogaster*, *MSA* is annotated as a variant of the *iab-8 lncRNA*. We know from colleagues that the *iab-8* transcript seems to be expressed in *D. hydei* accessory glands, which is probably the *MSA* version as in *melanogaster*. We are currently attempting to confirm this and explore its possible function in other *Drosophila* species experimentally by making reporter lines in *D. hydei* based on a conserved region around the predicted *MSA* promoter/enhancer. Although the transgenes have now been injected into *D. hydei*, they are not yet at a stage to be analyzed (*hydei* have a longer generation time and are proving difficult to work with). In any case, however, our repression mechanism mostly concerns *iab-8*, which seems to be conserved, at least in *Drosophila*.

5. "we showed that the MSA transcript serves as a template for a small peptide of 11-20 amino acids that we call MSAmiP". What is the exact length of MSAmiP? Is it 11 or 20 amino acids, or any value between 11~20?

Actually, this should have read 9-20 and we apologize for the error. We have actually removed a reference to the size from the text. However, for your information, in one of our previous works, we showed that there are three start codons in the *MSAmiP* sequence that can be used to initiate translation (Immarigeon et al. 2021). They would form peptides of 3, 9 and 20 amino acids respectively. Our fusion protein analysis indicated that while all three peptides may be made, the 9 amino acid-peptide seemed to be the most abundant. However, since we could not rule out the 20 amino acid form as also being important, we generally say that the peptide is 9-20 amino acids. Perhaps in the future, it would be better to say 9 or 20 amino acids.

Reviewer #2: In their manuscript "Upstream open reading frames repress the translation of a micropeptide involved in sperm competition" Maeda and colleagues explored the genetic regulation of the msa micropeptide (MSAmiP) from Drosophila melanogaster. This micropeptide is not produced in the central nervous system even though the iab-8 lncRNA transcript contains the MSAmiP sequence and is expressed in this tissue. The authors successfully dissected important sequences in exons 1 and 2 of the iab-8 lncRNA transcript that allow for the repression of the GFP fluorescence of a GFP tagged MSAmiP reporter gene and show that upstream ORFs are required to disrupt the fluorescence of GFP in different reporter genes by using S2 cells. Finally, they showed that exons 1 and 2 are sufficient to disrupt the fluorescence of a GFP reporter in vivo, and that mutagenizing the start codons in these exons allows GFP to be properly expressed.

Overall, the authors provide good evidence for their claims with a few caveats mentioned below. However, the main concern is that the in vivo relevance of the regulation is limited, and concerned to UAS constructs. Because they have a genetic engineering platform, I wonder if it is feasible to show that these upstream exons, or indeed specific ATG, are truly responsible to prevent MSA peptide expression in CNS. And, is MSA peptide expression in CNS deleterious? (E.g., is this regulatory mechanism necessary?)

Yes, we would love to show this, unfortunately, we can find no overt phenotype associated with the removal of these uORFs outside of a slight derepression of ABD-A. We also know that overexpression of *msa* does not seem to have any deleterious effect on fly development or fertility. Although this was mentioned in the previous version, it was perhaps not clear. We have tried to make this point clearer in this version. In any case, although we believe that the uORFs will regulate MSAmiP translation, it may not be the main reason for these uORFs. Instead, we propose that repressing unwanted ABD-A protein may be the main reason for these uORFs. Like many redundant safeguard mechanisms, this may not show a strong phenotype when modified by itself. We explore this a bit more in the updated discussion.

- When analyzing ORFs upstream of exon 2 by themselves (sections A,B,C,D and E in Figure 3) it should be noted that even if they can’t repress GFP in this assay, it is possible that they could still be repressive of the MSAmiP in vivo, since it could be that there are ORFs transcribing with GFP in frame and forming a longer protein that can still fluoresce but in vivo there might be a stop codon downstream that also inhibits the translation of the MSAmiP. Taking this into consideration and adding this information to the iab-8 lncRNA models is crucial to differentiate between translation inhibition due to an upstream uORF and a disruption of the MSAmiP due to the formation of a frameshifted MSAmiP.

After reading over this comment, we believe that this would apply to the C and E fragments shown in Figure 3. In these cases, it would be possible that there is an in-frame fusion between the start codons in the fragment and GFP. Checking the sequences, we can eliminate the start codons in fragment C because they are either followed by a downstream stop codon before GFP (the first start codon) or are actually incomplete (the last start codon is made at the junction between exons 1 and 2). For the E fragment, we can indeed find a possible in frame fusion. However, in the context of the full length *iab-8 RNA*, it is followed shortly by an in-frame stop codon present in Fragment F (see figure 4). Thus, these ATGs should not make MSAmiP fusion products. However, it is true that they may be inhibitory of downstream translation. Given that we do not claim to identify all repressive elements and because clarification of this point might lead to further confusion, we did not add this possibility to our discussion.

- Referring to Figure 7 the authors mention they were “not able to convincingly quantify this derepression for statistical analysis due to the variability in the staining procedure that resulted in changing background levels” if this is the case, they should include at least a few images of each condition to show the variability so that the reader can make their own conclusions.

Thank you, we have done this in the supplementary figures (S3).

- This may seem like a minor comment, but I believe it is a substantial issue. The figures are very difficult to read and understand. Fig1-5 are kind of confusing layout, it is a challenge to understand how the different reporter constructs and regions in different figures relate to each other, and what the construct names reflect. The immunostainings do not have even the markers labeled (Fig7). As a reader, this made it very difficult to understand their findings and conclusions.

We apologize for the difficulties in reading the manuscript. We have rewritten large portions of the manuscript and redrawn many of the figures, attempting to simplify representations without losing the information. We hope you find this version satisfactory.

Other minor comments about the manuscript:
- The 30 bp fragment of exon 1 is not mentioned in the text but has prominent activity change in Figure 1.

Yes, the 30 bp fragment does allow for GFP expression. As this assay focuses on the prevention of GFP expression, we used this only as a control to eliminate this area from the analysis of the 230 bp fragment.

- Authors say about the results in Figure 5C “Based on this, we hypothesized that, as a rule, upstream ORFs (uORFs) might repress downstream ORFs.”. This is one general proposal for uORF function, but they are writing it in a way that it is not quite meaningful. I think they are suggesting a competitive impact of translational initiation at a downstream main ORF, if the uORF is translated. But there could be other models at play, and this is not a biochemical mechanistic study. So I suggest to rephrase this.

Thank you for the suggestion. We have modified this area to say “… we hypothesized that, as a rule, translation of upstream ORFs might repress translation initiation from downstream ORFs. Thus, in B_small_, initiation from the first start codon would repress translation from the later start codons, but removal of an upstream start codon, as seen by dividing B_small_ and B_big_, allows for translation initiation to start at the downstream ATG sequence…”

- When showing fluorescence the Y axis should be more descriptive of what this fluorescence is (per cell, per slide, per pixel, etc). At least it should say “Fluorescence” instead of “Fluo”.

Sorry, this slipped by our editing and has now been corrected.

- The order of Figure 5B and 5C should be swapped for readability

We have modified this figure and hope that it is easier to understand.

Reviewer 3:

**Summary**

A vast body of work recognizes the physiological importance of so-called long non coding RNAs (lncRNAs), but recent studies have revealed that these lncRNAs can actually encode for micropeptides (miPeps) in addition to microRNAs (miRNAs). This work by Frei et al demonstrate that intricate translation regulation governs the expression of miPeps, and that such regulation has an outsized impact on physiology. The study investigates the translation regulation of the male-specific abdominal (MSA) transcript in *Drosophila*, which presents as two splice isoforms, one capable of encoding a micropeptide (MSAmiP) and another splice variant (called iab-lncRNA) incapable of doing so despite bearing the MSAmiP coding region. MSAmiP is produced in the secondary cells of male accessory glands and has been shown to be an important component of male seminal fluids, important for inducing postmating responses in females. The mechanisms underlying the seeming suppression of MSAmiP translation off the iab-8 lncRNA is unknown.

The authors use an extremely thorough array of control and mutant transgenic reporters in S2-cell based assays to investigate the upstream 5’ elements of iab-8 lncRNA. Their results reveal a prominent role for upstream open reading frames (uORFs) present in exons 1 and 2 (which are unique to iab-8 lncRNA and absent in the other MSA mRNA variant) in the suppression of MSAmiP translation. They break down these exons in careful 5’ “bashing” studies to show that translation initiation off a competent AUG in exon 1 acts as the primary suppressor of MSAmiP production off the iab-8 lncRNA. They also perform bioinformatic analyses across 44 different Drosophilid species to show the conservation of this ORF across evolutionary scales. The paper concludes with in vivo evidence where mutation of the relevant exon 1 sequences in the iab-8 lncRNA now results in spurious MSAmiP production. Overall this paper does a careful analysis of the translational regulation of the MSAmiP peptide which is important for fly fertility and establishes a necessary precedent in the study of lncRNA biology. The data in the paper are compelling, and the conclusions thus warranted. Regrettably, the paper suffers from inconsistent writing and confusing discussion in some parts. Barring heavy editing required to improve the readability of the paper and some minor clarifying experiments, I am fully in support of publication of this work. I’ve detailed my editorial suggestions and a couple of experimental concerns below.

Thank you for your careful reading of the manuscript and your support of its publication in PloS Genetics.

1. In Fig6E, the wing discs appear to be of different sizes. Are the larvae age-matched or is the difference in wing size a phenotype? Also, overexpression of UAS-exon1&2dATG appears to have an effect on tissue health in the accessory gland, is this tissue specific? Compartment specific overexpression of the same shows increased total wing size, or is the representative image simply from a slightly different developmental stage? Please clarify in text.

The slight differences in size and shape that the reviewer points out are artifacts of the staining procedure and we did not notice any differences in the discs or glands that could be associated with the change of genotype. The changes in the AG are due to the fact that the glands are essentially sac-like. After fixation, staining, and mounting, the glands can become “shriveled” to some degree. We believe much of this happens after changing buffers. With regards to the discs, we dissected discs of wandering third instar larvae. At this time, the discs are about to start the process of metamorphosis. It is hard for us to know exactly the reason for the disc to disc variation but it could be due slight differences in age or malformation due to tissue crowding during our fixation protocol (where we fix inverted whole larvae so as to prevent direct damaging the disc during dissection or losing the discs during subsequent washing). Looking over the slides, there was no effect that could be tied to the genotype. This is now explained a bit in the figure legend.

2. The wing disc images suggest incomplete penetration of the DAPI dye - please explain why the images only show DAPI staining on the margins of wing imaginal discs, and not within the notum and pouch.

Yes, we included the DAPI only to show the outline of the disc because black pictures (lack of GFP) are hard to interpret. We stained with DAPI by having DAPI in the mounting media. Dr. Frei, like many of us, took his images very soon after the mounting and DAPI was mostly seen in the cells facing the mounting agent. With the difference in staining between the outer cells and the inner cells, leveling the fluorescence levels was impossible. We have retaken the images of the same discs, which have now had more than enough time to equilibrate the DAPI levels.

3. The authors genetically manipulate accessory gland by ectopic reporter expression (Fig. 6) and by mutating the iab-8 lncRNA. However, there is very little clarity on the effects of these genetic manipulations on fertility, mating status, post mating response etc. It would be helpful to describe these phenotypes to fully appreciate the role of translation regulation of MSAmiPEP in male fertility.

Basically, removal of the iab-8 lncRNA is sterile due to the loss of the iab-8 miRNA. Indeed, we know that removal of the miRNA alone is sufficient to obtain this phenotype, and is the result of a neuronal problem where males become unable to properly bend their abdomen and females are unable to properly expel their eggs (see references to Bender 2008, Stark et al. 2008, Ronshaugen 2005 and Gummalla et al. 2012, and Gaurulet et al. 2014). Loss of the MSAmiP only results in a change in sperm competition (Immarigeon et al. 2021). As mentioned in this work, we could not detect any problem with the overexpression of the MSA transcript (which makes the MSAmiP peptide). We have tried to make this clearer in the current manuscript. We actually believe that this mechanism is probably an insurance mechanism to prevent unwanted ABD-A translation (see text). However, like many buffering mechansims, the effects are subtle.

4. The strongest physiological relevance for this study comes from their experiments described in page 16 paragraph 2, where the endogenous exon1/2 is replaced with a mutant one “lacking uORFs”. It is unclear what the authors mean by “lacking uORFs” especially in the milieu of all the uORF mutants they use in the paper. The authors describe that these exon1/2 mutants essentially act as iab-8 lncRNA nulls and show male sterility. However, no data is presented for any of these claims? Specifically no control is shown that splicing of the normal MSAmiPep-encoding MSA mRNA is unaffected by the mutation in exons 1/2. Similarly, it is unclear if the mutations in exon1/2 have any impact on miRNA generation off of the iab-8 lncRNA template.

We are sorry about the misunderstanding here. By lacking uORF, we mean that the area containing the two ORFs is deleted. We have now changed this in the text, mostly referring to removal to the two primary uORFs. Also, I think that there was a misunderstanding regarding the sterility phenotype. Loss of the iab-8 lncRNA is sterile. We know that our platform, where we deleted much of the promoter and first two exons of iab-8, is also sterile. In these experiments, we used, as a wt control, a line where we reintegrated the missing area back into its normal location. These flies are fertile and show no overt phenotype. When we make the ORF mutants, we essentially integrate the same DNA, just with the ORFs (approximately 300 bp) removed. These lines are also fertile and show no overt phenotype (except perhaps a very slight derepression of ABD-A in the posterior CNS). We hope that this is better explained in the new version of the text.

Editing recommendations

As it stands the manuscript has a ‘results and discussion’ section - for the sake of readability and elaboration, as well as conforming to journal standards, it would be very useful if the authors separated the sections.

Thank you, we have changed the text accordingly and also expanded the discussion in line with another reviewer.

The references to figures after description of the data is inconsistent throughout the manuscript. *e.g,* in some places, discussion of the data begins with “as see in figure xxx” (page 12, end of second paragraph), some places don’t reference a figure number (page 12, penultimate line). I would recommend consistently referencing the relevant figure for each data and discussion point throughout the manuscript to enable the reader to follow the authors’ rationale.

Thank you, I hope that the new text is better in this aspect. We apologize for the slip ups in editing, where different versions of the edited texts get spliced together and things slip by our tired eyes.

Please ensure consistent labeling across figures, especially of the genotypes and fluorescent markers, particularly Figure 7.

Thank you, we have tried to simplify the figures somewhat based on other reviewer comments. We hope to have a more consistent labeling.

The images in figure 6 and S2 (among others) are of lower resolution with barely readable text. Hopefully this will be resolved for publication.

Thank you, as I mentioned above, the figures have all been redrawn and we hope are now more easy to read.

Page 16 paragraph 2: s/spiced/spliced

Thank you for catching this mistake.

Fig 1.b - Y axis label reads ‘Fluo’ - it can be expanded to say ‘GFP fluorescence’

Again, thank you. We have changed all labels to Fluorescence (arb. unit) for arbitrary units (explained in the legends).

Ensure that *Drosophila* is consistently italicized across the text.

Thank you, we have done this.

The titles of the figure legend should reflect the key finding reported in that figure instead of a brief description of the experiment performed.

Thank you, we have done this.

In the figure legends, please be consistent and ensure the sub figures are labelled (for example, Figure 1B is simply mentioned as ‘the bottom panel’ but A. is mentioned in the legend. Some figure legends also have the sub figure letter in bold (fig 1A in the legend) but not in others (fig. 2A in legend). This is also confusing to read since figure 3 has fragments labelled as ‘A,B…F’ but the legend makes no mention of the sub figure labels. Please correct this appropriately to make it easier for the reader to understand the study.

Thank you, we have tried to make more complete and consistent legends.

DAPI is all capitals since it is an abbreviation, please correct in text and figures.

We apologize for our oversight.

Page 8, “mixes of plasmids were mixed” is redundant.

Yes, it is.

In figure 3, the authors divide up exons1/2 into A-F regions, which is quite helpful in navigating this complex mRNA. However, it would serve them well to introduce this method of representing the exons1/2 prior to the data in Fig. 2.

Thank you, while we have not done this because we thought it easier to explain a simple experiment before taking on the whole exon 1 and 2 region, we have tried to simplify the text and figures to help make the manuscript easier to read and follow.
